# Signal quality of simultaneously recorded endovascular, subdural and epidural signals are comparable

**DOI:** 10.1038/s41598-018-26457-7

**Published:** 2018-05-30

**Authors:** Sam E. John, Nicholas L. Opie, Yan T. Wong, Gil S. Rind, Stephen M. Ronayne, Giulia Gerboni, Sebastien H. Bauquier, Terence J. O’Brien, Clive N. May, David B. Grayden, Thomas J. Oxley

**Affiliations:** 1 Department of Biomedical Engineering, The University of Melbourne, Parkville, Australia; 2 Vascular Bionics Laboratory, Department of Medicine, Royal Melbourne Hospital, (RMH), The University of Melbourne, Parkville, Australia; 3 Florey Institute of Neuroscience and Mental Health, Parkville, Australia; 4 Centre for Neural Engineering, The University of Melbourne, Carlton, Australia; 5 Department of Veterinary Science, The University of Melbourne, Werribee, Australia; 6 Department of Physiology and Department of Electrical and Computer Systems Engineering, Monash University, Clayton, Australia; 7 SmartStent Pty Ltd, Parkville, Australia

## Abstract

Recent work has demonstrated the feasibility of minimally-invasive implantation of electrodes into a cortical blood vessel. However, the effect of the dura and blood vessel on recording signal quality is not understood and may be a critical factor impacting implementation of a closed-loop endovascular neuromodulation system. The present work compares the performance and recording signal quality of a minimally-invasive endovascular neural interface with conventional subdural and epidural interfaces. We compared bandwidth, signal-to-noise ratio, and spatial resolution of recorded cortical signals using subdural, epidural and endovascular arrays four weeks after implantation in sheep. We show that the quality of the signals (bandwidth and signal-to-noise ratio) of the endovascular neural interface is not significantly different from conventional neural sensors. However, the spatial resolution depends on the array location and the frequency of recording. We also show that there is a direct correlation between the signal-noise-ratio and classification accuracy, and that decoding accuracy is comparable between electrode arrays. These results support the consideration for use of an endovascular neural interface in a clinical trial of a novel closed-loop neuromodulation technology.

## Introduction

The endovascular neural interface, known as the Stentrode^TM^, provides a minimally-invasive method for recording brain signals and, potentially, stimulating cortical tissue without the need for risky, open-brain surgery^1^. Methods to achieve endovascular brain recordings have progressed significantly from the earliest use of a wire in a cerebral blood vessel^2^, to a catheter mounted device^3,4^ and recently to the development of the Stentrode device^1,5^. Chronically-implantable endovascular devices are a promising method to achieve brain recordings without the need for craniotomy.

The minimally-invasive nature of implantation makes the endovascular (EV) approach desirable for use as a brain-machine interface (BMI). Previous applications of BMI based on cortical surface brain recording have used subdural (SD) arrays, which are placed under the dura, or epidural (ED) arrays, which are placed above the dura^6–13^. Despite many successful studies^9,14–16^, SD and ED devices require a craniotomy for implantation and are associated with a risk of infection, surgical complications, and mortality^17^. The EV array avoids the use of a craniotomy while still recording surface potentials from the brain. However, the clinical significance, quality, and efficacy of signals recorded is not clearly understood.

EV neural interfaces are delivered to the target area via cortical blood vessels using concentric catheters^1,3–5,18–26^. Studies have shown that neural signals can be recorded from microwires, catheter mounted electrodes, wire mounted electrodes, or stent mounted electrodes. A thorough review of endovascular technology was provided by Sefcik *et al*.^5^. The first EV neural interface consisted of a 0.6 mm electrode mounted on a guidewire tip^2^. This was followed by several short reports of similar recordings with guide wires^3,5,27–33^. The next major advance was two decades later, also recording with a microwire^31^, closely followed by a landmark study showing the feasibility of a multi-channel EV array with 16 electrodes^3^. Another recent study in the field showed recordings from a nanowire electrode array (0.6 µm diameter) in a capillary^32^. Most studies prior to 2016 were performed in humans undergoing surgery or in animal models acutely and therefore only lasted a few hours. The next major study was published in 2016 with electrodes mounted on a self-expanding stent^1^. This study was the first to show the ability to chronically implant a stent mounted electrode array into a blood vessel and record neural information over periods up to 6 months. Endovascular technology has progressed significantly from the earliest use of a wire in the brain to record brain signal to catheter mounted devices and now stent mounted devices^5^. In the last 10 years, there have been few reports of brain signal recording from acute implantation of catheters or wires in a cortical blood vessel^4,18,20–22,33^. Two studies, He *et al*.^18^ and Bower *et al*.^4^ also evaluated the signal quality of the recordings using electrodes acutely placed in blood vessels in acute implantation lasting a few hours and added significantly to the field.

Bower *et al*.^4^ showed for the first time that microelectrodes (40 µm diameter) could record cortical signals from within a blood vessel. Using a porcine model, a catheter-based electrode array was placed into the superior sagittal sinus (SSS) via a small incision in the vein. High amplitude spikes (>0.5 mV) were generated using penicillin injections into the cortex and acute recordings from an endovascular catheter array (macro ring electrode and micro disc electrodes) were compared to subdural arrays. The authors noted that epileptiform spikes from 40 µm disc electrodes and a 1 mm wide ring (other dimensions not reported) placed endovascularly had the same amplitudes as 40 µm and 2 mm disc electrodes placed subdurally. They also noted spatially localized ‘microspikes’ recorded by the SD and EV microelectrode arrays, but not on the macro arrays. However, the paper did not quantify the SNR of the recording or the spatial resolutions obtainable by the electrodes. Typical oscillations in the brain recorded by subdural and epidural arrays range in the order of 10–500 uV^34^ while, during an epileptic event, the synchronized high amplitude signals may not be easily differentiated. The high amplitude spikes would also make it difficult to quantitatively differentiate recording properties between electrode sizes or the effect of the tissue surrounding the electrodes. It is noteworthy that similar qualitative patterns were noted on both microarrays that were different from macroarrays. The study showed the feasibility of electrodes within a blood vessel in recording epileptiform spiking, leading to the conclusion that endovascular arrays would be useful in recording neural signals toward localization of epileptogenic foci.^16^

He *et al*.^18^ used a guidewire electrode similar to previous studies^5^ in a porcine model and showed that guidewire electrode recording quality defined by the SNR of auditory and visual evoked potentials was superior to (scalp) electroencephalography (EEG). The comparatively superior SNR of guidewire electrode recording was not surprising as the skull is a strong attenuator of cortical signals and the guidewire electrodes were under the skull. It is more important to evaluate whether endovascularly placed electrodes are comparable to SD and ED electrodes^1,4^ which are all implanted under the skull and closer to the brain than EEG. He *et al*. found a dependence of SNR on location of the wire; however, the spatial resolution was not quantified. Interpolation of the figures appears to indicate spatial resolution in the order of 10’s of millimeters, though it would be expected that the spatial resolution of endovascular electrodes would be close to that of subdural and epidural arrays in the order of 2–6 mm.

While both Bower *et al*.^4^ and He *et al*.^18^ showed some quantification of signal quality, both studies used catheters or guidewire mounted electrodes to perform recordings^5^. Furthermore, both studies show recordings over an acute implantation period of a few hours. Until now, only Oxley *et al*.^1^ has demonstrated a stent-based device that can be chronically implanted into the blood vessel and record neural activity. Furthermore, a chronic six month study^1,23,25^ used electrodes opposing the blood vessel wall, which showed that the process of incorporation of the electrode takes approximately 14 days and recordings before this time were highly variable and, in some cases, not differentiable from noise. To date, the stent mounted technique is the most feasible technique for chronic implantation and recording, making it possible to envision multiple applications of the minimally invasive EV neural interface^1,5,35^.

In our previous work^1^, we compared the signal bandwidths and artefacts of the first generation chronically implantable stent-based EV device with macro SD and ED electrodes. The SD and ED electrodes used in the study were much larger than the EV electrodes, leading to a skewed view toward the larger electrode sizes. In this previous work, we alluded to the potential of high spatial resolution with the EV arrays, but this was not evaluated. Furthermore, the SNR and the effect of noise on the signals were not investigated. Further work is required to understand the clinical utility and to establish comparability to conventional electrodes to be considered as a feasible method neural interfacing.

**Figure 1:**
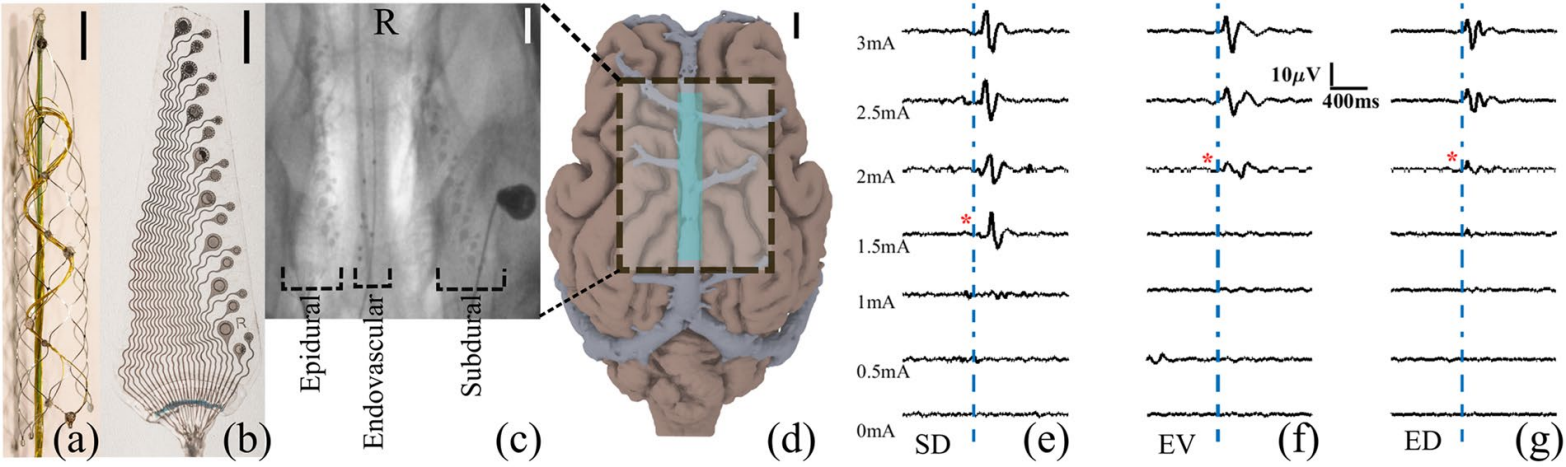
Implanted devices, their placement, and example recordings. (**a**) EV array. (**b**) ED/SD array (Cortec gmbh, Freiburg, Germany). (**c**) X-ray image of the ED array (left), EV array (middle, implanted in the superior sagittal sinus), and SD array (right) implanted in a sheep brain. R, rostral. (**d**) MRI reconstruction of the sheep brain with the major veins shown. The box region shows the implantation site of the arrays, with the superior sagittal sinus marked in green. All scale bars are 5 mm. Averaged electrically evoked potentials were obtained by stimulating the median nerve at different current levels using cathodal monophasic constant current pulse, while simultaneously recording from the (**e**) SD, (**f**) EV, and (**g**) ED arrays. Red * indicates threshold level of stimulation.

In the present manuscript, we evaluate the SNR and the ability to detect a signal with the EV array (Fig. 1a) in comparison to SD and ED arrays (Fig. 1b). Measurements were made three weeks after implantation, providing sufficient time for incorporation of the devices into the blood vessel wall. In all previous studies except Oxley *et al*. measurements were made within minutes/hours after implantation and, therefore, prior to incorporation of the electrodes into the tissue. Efficacy of brain recordings, such as those obtained using EV, SD, or ED arrays, can be characterized by the recording bandwidth, signal-to-noise ratio (SNR), and spatial resolution. The bandwidth provides an estimate of the maximum frequency range over which neural information can be used for useful interpretation of the brain signals. The SNR is a critical feature of any clinical neural interface as it has a strong correlation with decoding performance for a BMI^36–38^. The spatial resolution achievable with any given device demonstrates its ability to record spatially localized activity. Arrays with better spatial resolution can record spatially specific information that is vital in accurately decoding movement intent in a BMI^36,39,40^.

In the present study, we systematically investigated the effects of electrode size and location on the signal bandwidth, sensitivity, SNR, spatial profile, and ability to decode the recordings. We compared the signal quality of recordings obtained with EV arrays to those from conventional SD and ED arrays implanted in sheep. The results demonstrate that that the EV array is a suitable candidate to decode neural information that may be used in a BMI.

## Results

Figure 1 shows the electrode arrays used in this study (Fig. 1a,b), placed in the superior sagittal sinus of sheep (Fig. 1c,d). Example recordings during median nerve stimulation are shown in for SD, EV and ED arrays in Fig. 1e–g respectively. The red asterixis shows where a discernible response to the stimulation of the median nerve was detected. The evoked potential waveforms of Fig. 1(e–g) showed differences in the waveform shapes when visually assessed. The differences in the shapes were not consistent across all animals and, since electrode positions across experiments varied in each animal, the waveform shapes were thus not assessed. However, waveform shapes may hold additional information regarding the underlying neural of the response and would be better suited to be addressed in an animal model that is better understood, with comprehensive literature and understanding of the structure and function of cerebral cortex.

### Comparison of bandwidths of SD, EV and ED arrays

**Figure 2:**
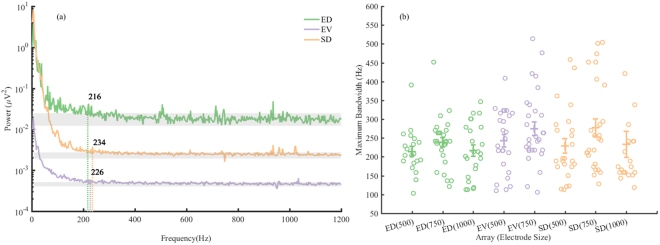
Electrode bandwidths. (**a**) Frequency spectra from representative 500 µm SD, EV, and ED electrodes, displaying characteristic (1/f) frequency responses. Band powers were calculated in individual 2 s windows using the Thompson multitaper method with a centre frequency of 1 Hz (2 Hz resolution). Dashed vertical lines and numbers indicate calculated maximum bandwidths. Grey bars indicate respective noise floors. (**b**) Maximum bandwidths for ED, EV, and SD arrays for electrode sizes 500, 750, and 1000 µm. Circles show individual values, centre lines show mean values, error bars show standard error of the mean. Two-way ANOVA showed no significant effect of either the array location (p = 0.75) or electrode size (p = 0.15) on the bandwidth.

The bandwidth of surface local field potentials (LFP), such as those that are recorded by the SD, EV, and ED arrays, have been reported to be less than 500 Hz^1,34,41^. The limit of the amount of information that can be recorded is thought to be related to the distance between the recording electrodes and the target neurons and to the sizes of the electrodes^36,37^. The bandwidth of recorded signals provides an estimate of the quantity of information that can be obtained using the SD, EV, and ED arrays. Figure 2a shows frequency spectra and bandwidth estimations from representative electrodes of the three arrays from baseline recordings in awake, resting animals. The power spectra in Fig. 2a show that the differences in the powers were evident across all frequency bands and not limited to the frequencies representing the noise. The raw power spectra were not normalized for comparison of the maximum bandwidth.

Figure 2b shows bandwidths measured from each array and with each electrode size. Bandwidth from all arrays were normally distributed and showed large variability in bandwidth across electrodes. Two-way ANOVA showed no significant effect of either the array location (F(1,147) = 0.1, p = 0.75) or electrode size (F(1,147) = 2.08, p = 0.15) on the bandwidth. There was no significant interaction between recording location and size of electrode (F(3,147) = 0.38, p = 0.76). In our previous study^1^, the SD and ED electrodes were larger in size (4 mm diameter) than the EV (0.75 mm diameter), which possibly influenced the bandwidths recorded^42^.

### Single trial signal-to-noise ratios of SD, EV and ED arrays

**Figure 3:**
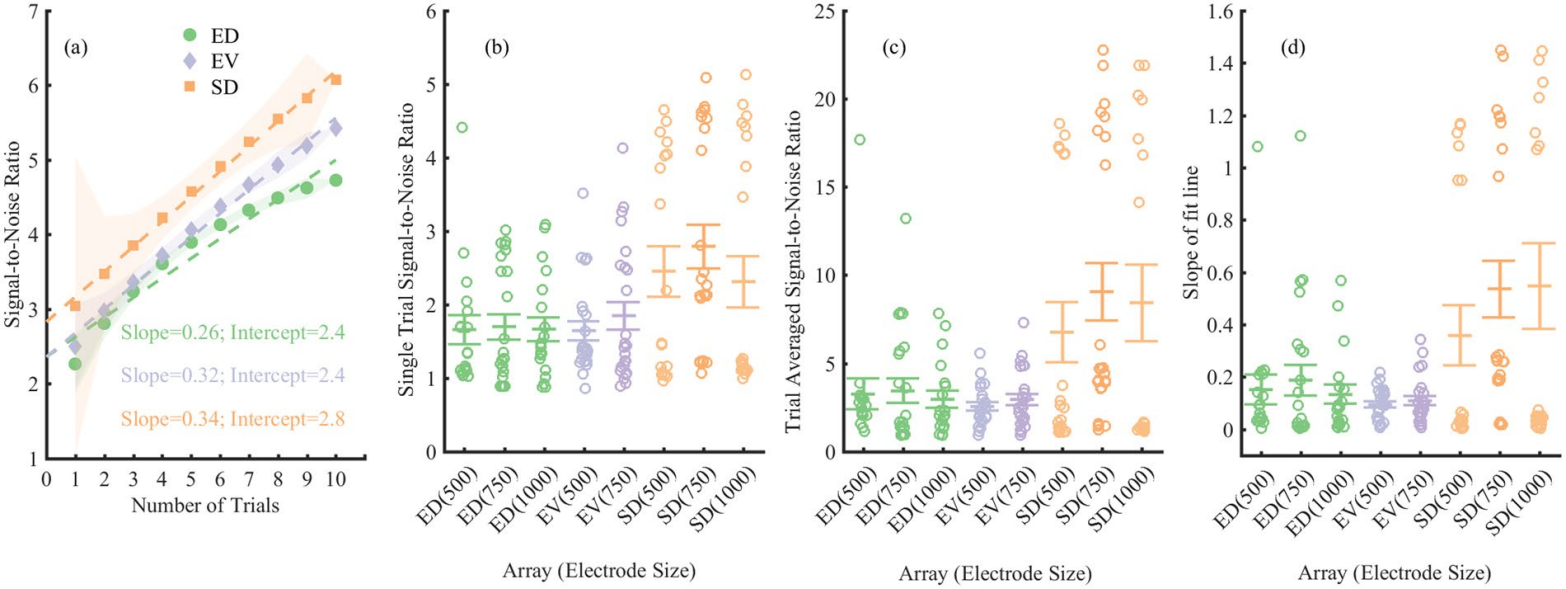
Signal-to-noise ratios (SNRs). (**a**) Example of SNRs vs. number of trials (repetitions of stimuli) for the ED (green), EV (purple), and SD (orange) 750 µm diameter electrodes. Example traces were taken from electrodes closest to each other as determined on an x-ray image. Shaded areas indicate standard error of the mean. The lines are straight line fits (y = P*x + Q) for each array, where x is the number of trials, P is the slope of fit line, and Q is the intercept. (**b**) Single trial SNR. (**c**) SNR averaged over 10 trials. (**d**) The slopes ‘P’ of the fit lines shown in (**a**). Symbols show individual values, centre lines show mean values, error bars show standard error of the mean. Mood median test showed no significant effect of electrode size or location on either; (**b**) single trial SNR, (**c**) Trial averaged SNR or (**d**) the slope of fit line (p > 0.05).

The efficacy of neural recording in detecting neural events and decoding activity is improved with greater signal amplitude relative to background noise. Therefore, signal-to-noise ratio (SNR) is a useful measure of signal quality, where SNR = 1 indicates equal signal and noise levels. For a BMI, it is important that the SNR is as high as possible to ensure recordings yield high decoding accuracy^43^. Figure 3a shows change in SNR of 750 µm electrodes with different numbers of trials included in the average response. Slope here shows the rate of change of the SNR with number of trials. Figure 3b shows the single trial SNR, Fig. 3c shows the SNR calculated on the signal after 10 consecutive trials were averaged together, and Fig. 3d shows the slope of the fits in Fig. 3a for all arrays and animals.

The data were not normally distributed with differences in standard deviations and varying distribution shapes; therefore, a Mood median test was performed to quantify the effect of electrode size and array location on the SNR. The single trial SNR (Fig. 3b) did not show a statistically significant effect of electrode size (χ^2^ = 2.17, DOF = 2, p = 0.33) or array location (χ^2^ = 1.79, DOF = 2, p = 0.40). Similarly, the trial averaged SNR (10 trials) in Fig. 3c showed the effects of electrode size (χ^2^ = 5.07, DOF = 2, p = 0.07) or array location (χ^2^ = 3.24, DOF = 2, p = 0.19) were not statistically significant. However, it should be noted that the SNR of the SD showed a wide range of values (interquartile range (IQR) = 15.89) with a bimodal distribution compared to the those of the EV (IQR = 1.71) and ED arrays (IQR = 2.45), which showed unimodal distributions. The wide range and bimodal distributions of SD-SNR values in Fig. 3b–d, indicate that some SD electrodes outperformed the EV and ED arrays. The rate of change of SNR (Fig. 3d) from 1 to 10 trial averages given by the slope also showed no significant effect of electrode size (χ^2^ = 2.63, DOF = 2, p = 0.26) or array location (χ^2^ = 0.14, DOF = 2, p = 0.93). Since the SD array is closer to the neural tissue than the ED and EV arrays, a higher SNR would be anticipated. The thickness of the dura in sheep measured 80–100 µm, with cerebrospinal fluid (CSF) separating the dura and the brain; in previous work, we showed the SSS vessel wall thickness varied from 200–600 µm^25^. The binomial distribution of the SNR of SD arrays may have also resulted from electrode locations where some electrodes on the SD array were closer to the source of the evoked potentials. The binomial distribution of the SNR of SD arrays may have also resulted electrode locations where some electrodes on the SD array were closer to the source of the evoked potentials. Previous work has also shown SD electrodes tend to have higher amplitudes than ED electrodes^42^. Our results indicate that, four weeks after implantation, the SNR of SD, EV, and ED electrodes were not significantly affected by electrode size. However, some electrodes on the SD array clearly outperformed the SNR of the EV and ED arrays, whereas other SD electrodes showed an SNR comparable to those of the EV and ED arrays.

### Spatial resolutions of SD, EV and ED arrays

The spatial resolution of an array refers to the ability of the array to localize discriminable neural signals. Spatial resolution is largely a function of distances between recording electrodes on an array, distances between electrodes and neural signals of interest, and electrode sizes. Higher spatial resolution provides greater specificity for a BMI^44^.

**Figure 4:**
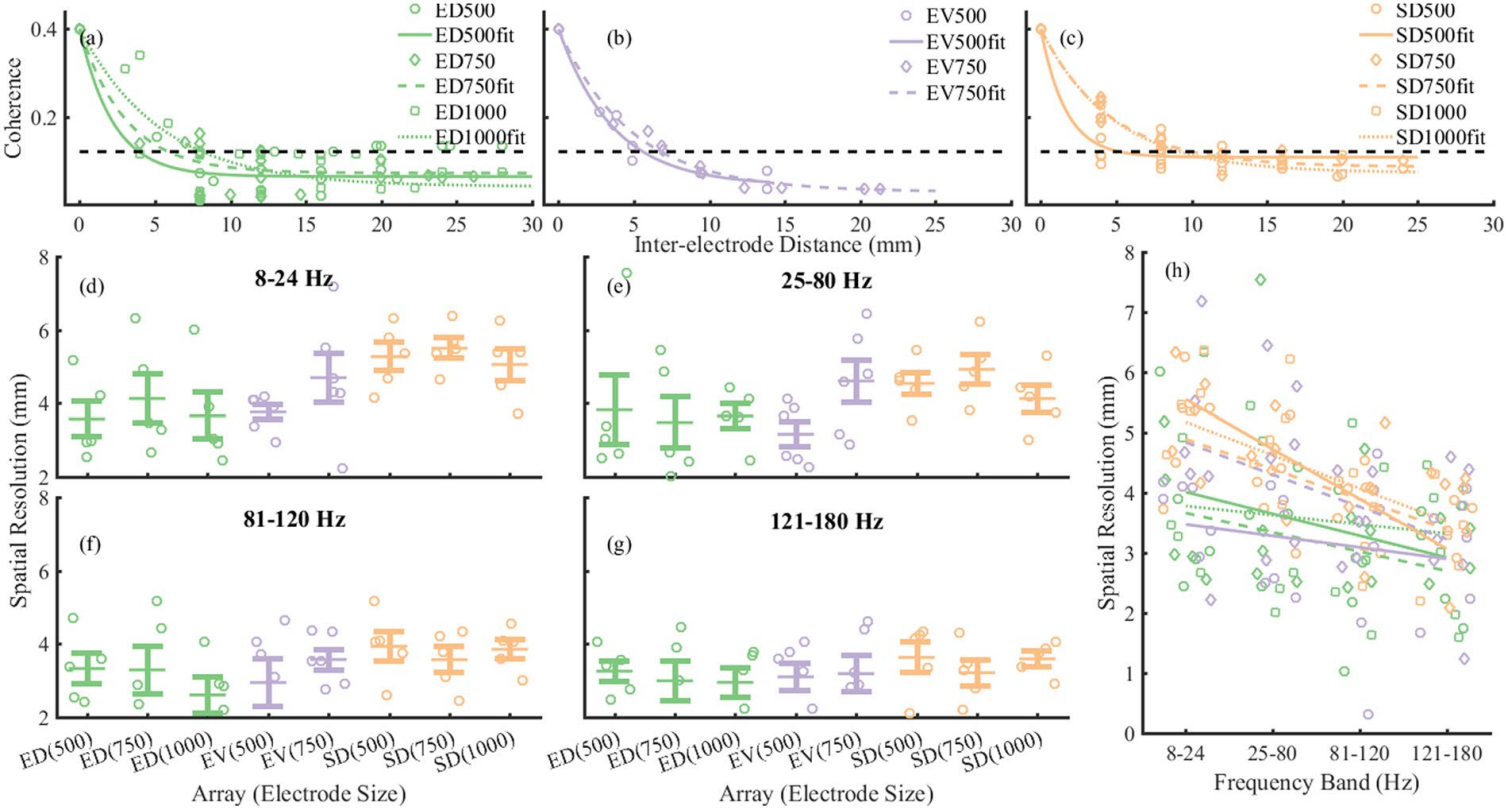
Spatial resolution. Representative data showing the estimation of spatial resolution using magnitude squared coherence versus the inter-electrode distance for (**a**) ED, (**b**) EV, and (**c**) SD electrodes. Fits were estimated as an exponential function of the magnitude squared coherence and were weighted to the inverse of the inter-electrode distance. The dashed horizontal line at 0.3 shows the level at which the signals between signals were considered independent. (**d**) Spatial resolutions at 8–24 Hz. (**e**) Spatial resolutions at 25–80 Hz. (**f**) Spatial resolutions at 81–120 Hz. (**g**) Spatial resolutions at 121–180 Hz. Symbols show individual values, centre lines show mean, error bars show standard error of the mean. Kruskal-Wallis test showed a significant effect of electrode location in the low frequency (**d**) (p = 0.003). However, there was effect of electrode size at any frequency band (p > 0.05) or electrode location at frequencies greater than 24 Hz (**e**–**g**). (**h**) Frequency dependence of spatial resolution, symbols indicate electrode size and lines are global fits at each electrode size and array. Pearson’s correlation analysis showed strong negative correlation between spatial resolution and frequency for all electrode sizes with SD arrays ρ > 0.6 (p < 0.05); moderate negative correlation 750 µm diameter EV electrodes ρ = 0.45 (p < 0.05); and weak correlations not significantly different to zero for ED electrodes and 500 µm diameter EV electrodes (p > 0.1).

Our results showed that spatial resolution was frequency dependent and was dominated by array location at lower frequencies but not by electrode size for electrodes between 500-100 µm in diameter. Figure 4 (a–c) show a reduction in the magnitude squared coherence with increasing inter-electrode distances for ED, EV, and SD arrays. The data have been fitted with exponential curves^39^. The dashed horizontal line at coherence = 0.3 shows the threshold level above which the data from the two electrodes were said to be arising from a common source.

Figure 4 (d–g) show the mean spatial resolutions for all the arrays at each electrode size in four frequency bands: 8–24 Hz, 25–80 Hz, 81–120 Hz, and 121–180 Hz. The data were normally distributed but unbalanced with a sample size limited to the number of animals – ED (N = 5 animals), SD (N = 5 animals), and EV (N = 6 animals), so we used a Kruskal-Wallis test to compare medians of spatial resolutions between groups. Spatial resolution measurements were repeated in the 8–24 Hz frequency band (Fig. 4d), the median spatial resolutions at different array location (SD, EV, and ED) was statistically significant (χ^2^(2) = 11.42, p = 0.003) but the median spatial resolutions did not vary significantly with effect of electrode size (χ^2^(2) = 1.28, p = 0.52). The median spatial resolution from EV electrodes varied least from the median of all groups (z = 0.45) while the median spatial resolution of the ED arrays was lower (z = −2.29) and the median SNR of the SD arrays was higher (z = 2.78) than the median of all groups.

In the 28–80 Hz frequency band (Fig. 4e), the median spatial resolution did not vary significantly with either the array location (χ^2^(2) = 5.83, p = 0.054) or electrode size (χ^2^(2) = 4.97, p = 0.083). Similarly, in the 81–120 Hz frequency band (Fig. 4f), median spatial resolution did not vary significantly with either the array location (χ^2^(2) = 5.63, p = 0.06), or electrode size (χ^2^(2) = 1.81, p = 0.40). Likewise, in the 121–180 Hz frequency band (Fig. 4g), there was no statistically significant effects of array location (χ^2^(2) = 2.22, p = 0.33) or electrode size (χ^2^(2) = 0.88, p = 0.64) on the spatial resolution.

Figure 4h shows the changes in spatial resolution with frequency for SD, EV, and ED arrays at all electrode sizes. Fit lines shown are global linear regressions for each electrode size in all arrays. Pearson’s correlation showed there was a moderate to strong negative correlation between spatial resolution and frequency, at all electrode sizes for SD arrays (500 µm, ρ (2) = −0.60, p = 0.005; 750 µm, ρ (2) = −0.78, p = 0 0.0001; 1000 µm, ρ (2) = −0.62, p = 0.003). There was also a moderate negative correlation between spatial resolution and frequency for 750 µm EV arrays (ρ (2) = −0.46, p = 0.02). There were weak negative correlations observed between spatial resolution and frequency at all electrode sizes for ED (500 µm, ρ (2) = −0.13, p = 0.56; 750 µm, ρ (2) = −0.30, p = 0.20; 1000 µm, ρ (2) = −0.33, p = 0.15) and 500 µm EV arrays (ρ (2) = −0.25, p = 0.25); however, these weak negative correlations were not significantly different from zero.

Results showed that the spatial resolution was frequency dependent and varied with the array location and to a lesser extent on the electrode size. At the onset of the study, it was expected that SD arrays would have the highest spatial resolution since the SD electrodes are closest to the brain and in contact with the cortical surface. However, there were minimal effects of array location on the spatial resolution at frequencies greater than 24 Hz in the resting state. In all three arrays, there was no effect of electrode size in electrodes between 500–1000 µm in diameters.

### Single trial decoding performance of evoked potential in SD, EV and ED arrays

The goal of BMI technology is to decode neural signals accurately to control external interfaces. Accuracy of control provides a measure of the reliability of the decoding. The accuracy of decoding is a key indicator of the ability to decode discrete user activity and is dependent on several factors. One key factor that could enhance decoding accuracy is the effect of the SNR on the signal. We therefore measured the accuracy of decoding discrete evoked potentials in sheep.

**Figure 5:**
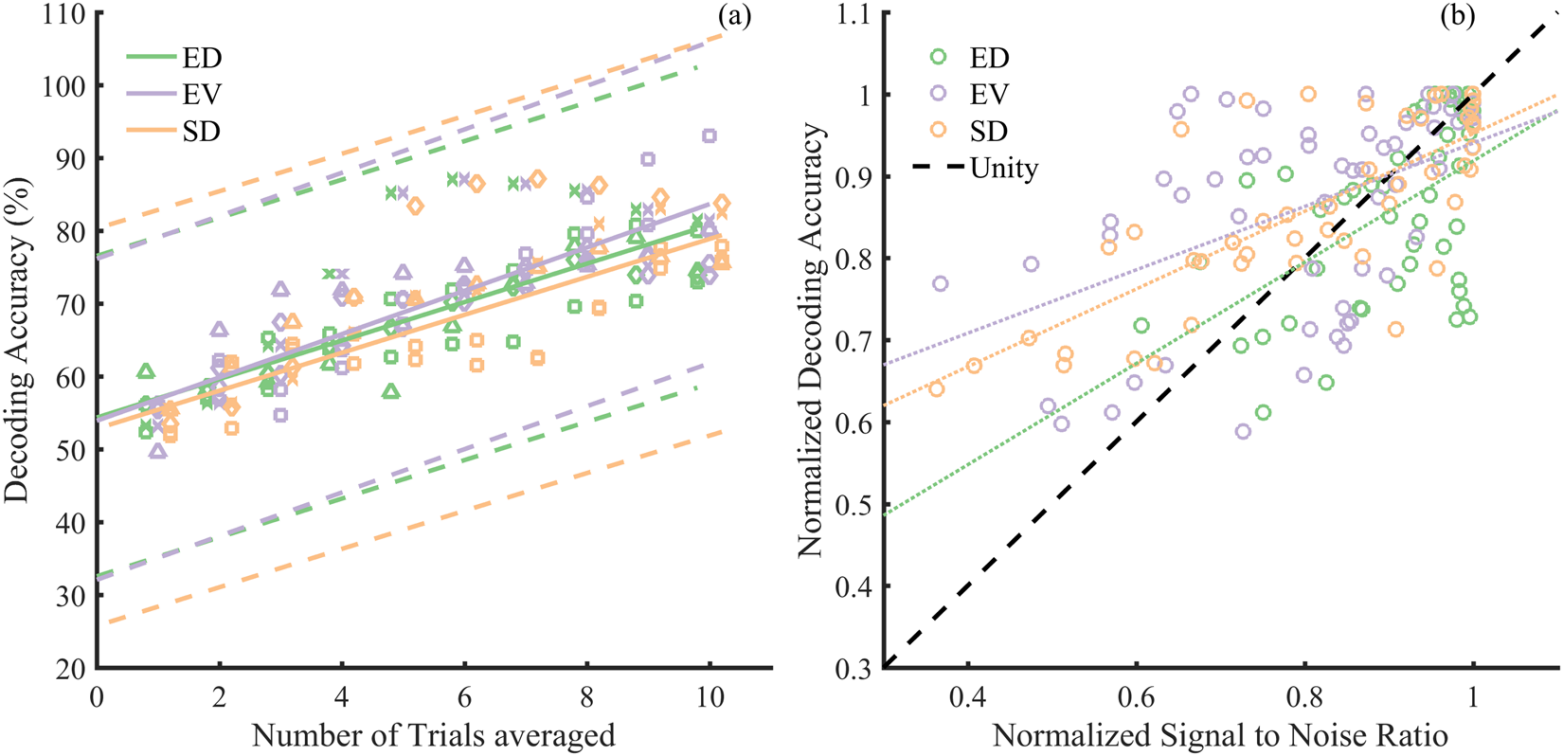
Decoding accuracies. (**a**) Decoding accuracy of evoked potentials versus number of trials used in calculating the average. Symbols indicate different animals and colours indicate the ED (green), EV (purple), and SD (orange) arrays. The fit lines are global fits on decoding accuracy across all animal; ED (N = 5 animals), SD (N = 5 animals), and EV (N = 6 animals) for each array. The symbols for each array have been adjusted in the x-axis direction to improve visualization. Dashed lines show 95% confidence intervals. Global Fit lines showed a good fit to all arrays r^2^ > 0.7. (**b**) Dependence of the decoding accuracy on SNR. Both decoding accuracy and SNR are normalized to the max of decoding accuracy and SNR, respectively, in each animal Dotted lines show global fits across all animals. Pearson’s correlation showed strong (ρ > 0.7, p < 0.05). correlation between SNR and decoding accuracy for all arrays.

Figure 5a, shows the dependence of the decoding accuracy in detecting an evoked potential on the number of trial averages for the SD (N = 5 animals), EV (N = 6 animals) and ED (N = 5 animals), arrays. A global linear regression applied to the decoding accuracy in Fig. 5a, from all animals for each array, showed the performances of the three arrays were comparable with overlapping confidence intervals. Slopes of the global fits were 2.642 (r^2^ = 0.77, p = 0.0001) for ED arrays, 2.97 (r^2^ = 0.74, p = 0.0001) for EV arrays, and 2.60 (r^2^ = 0.72, p = 0.0001) for SD arrays. Tukey corrected multiple paired t-tests between the accuracies of SD, EV, and ED arrays also showed no statistically significant differences in the decoding accuracies (p > 0.05 for all comparisons).

Figure 5b shows the dependence of the decoding accuracy on the SNR. Both SNR and decoding accuracy were normalized by their maximum value within each animal due to large inter-animal variability. There was a linearly increasing improvement in decoding accuracy with increasing SNR. Pearson’s correlation calculated for each animal showed a strong correlation [ρ (degrees of freedom)] in all arrays. Mean correlations ρ (2) (N, standard deviations) were: ED, ρ (2) = 0.77 (N = 5, σ = 0.393), p = 0.048; EV, ρ (2) = 0.80 (N = 6, σ = 0.11), p = 0.041 and SD ρ (2) = 0.92 (N = 5, σ = 0.03), p = 0.001.

Results showed that there were no differences in decoding accuracies from the ED, EV, or SD arrays. However, we found that there was a strong correlation between SNR and the decoding accuracy in all three arrays.

## Discussion

To date literature in endovascular electrocorticography has been sparse^5^. However, since 2016 there has been a steady increase in the literature in endovascular electrocorticography owing to the steady improvements in the technology^5^. Thus far previous work has shown that: endovascular electrodes may be placed in a cortical blood vessel to record and stimulate the brain^1,3,5,18–22,45,46^; signals quality is likely comparable to that of other intracortical arrays^1,4^; electrode size (micro versus macro electrode) affects the type of signal recorded (i.e. smaller electrodes <100 µm diameter can recording local field potentials)^4^. However there are notable discrepancy between studies^1,4,42^ relating the effects of dura on the signal recorded.

Here, we show that: (1) The decoding accuracy is comparable between the EV, SD and ED arrays; (2) There is a correlation between the signal-noise-ratio and classification accuracy in all three recording modalities; (3) Bandwidth spatial resolution and signal to noise ratio of recordings from the EV arrays are comparable to SD and ED recordings; (4) The dura does not significantly reduce the signal to noise ratio; and (5) SD arrays had the best spatial resolution of the three arrays only at frequencies below 25 Hz. These results extend prior work by comprehensively addressing equivalence of EV recordings to standard ED and SD arrays with regard the bandwidth, signal-to-noise ratio, the spatial resolution and decoding ability of these devices for potential BCI applications.

Neither the dura nor the blood vessel significantly affect signal quality or performance. While some differences were observed in the signal waveform and the signal powers between electrodes on the SD, EV, and ED arrays, the SNR was similar across the three arrays; i.e., the absolute power was smaller but not the SNR. This indicates that the noise and the signal changed proportionally in the three arrays. For example, the EV array showed the lowest raw power, resulting from the low signal amplitudes seen on the EV array; however, the SNR was comparable with the SD and ED arrays. Contrary to some literature^42,47,48^, our results indicate that there was negligible effect of dura or blood vessel on the quality of the signals. This was also seen in the decoding performance, where there were no significant differences between ED and SD recordings^49^. Bower *et al*.^4^ also showed a similar result where there were no discernible differences between SD and ED electrodes. The finding that there was no discernible difference between SD and ED was surprising as the SD arrays which are closer to the brain than the ED arrays. The ED similar to the EV array is separated from the brain by the dura and CSF. In addition, the EV array has an additional layer of the blood vessel wall in the case of EV. It would be expected that some differences in signals would have been evident that relate to the distance from the brain or the tissue. The result of Bower *et al*.^4^ may have been due to the large epileptiform spiking amplitudes (0.5–1 mV) used to assess the signal which are much greater than typical intracortical brain signals of 10–50 µV amplitudes. At these high amplitudes typical intracortical brain signals may not be discernible as by Bower *et al*.^4^. In the present study we showed that the electrode location affected the spatial resolution at low frequencies but there were no significant differences in the signal quality measured by the bandwidth, SNR and decoding ability. The lack of difference in signal quality in particular SNR of the three array types in the present study may be due to the time points at which signal quality was assessed. The time points used in this study are 25 days post-implantation for the EV array and 21 days post-implantation for the ED and SD arrays. Chronic histological studies have also shown that the immune response to SD and ED arrays results in an immediate fibrous tissue encapsulation occurring over 14 days, followed by long-term tissue responses^50,51^. The EV arrays are not encapsulated with a fibrous layer, instead, they are covered by a thin layer of endothelium^1,25^. It is reasonable to consider that fibrous tissue encapsulation in SD arrays would result in a migration of implanted electrodes further away from the surface of the brain. It is possible that the advantage of the SD array being close to the brain may be circumvented by the fibrous encapsulation post-implantation, making the SNR and bandwidth of the ED, SD, and EV recordings more comparable.

Previous work^47^ also noted that the dura itself did not change the spatial resolution but rather it was the thickness of the CSF that made the greatest difference. Furthermore, conductivity measures of CSF, endothelium and dura are higher than that of fibrous tissue^52,53^ such as would be expected to be encapsulated around SD and ED electrode. It can therefore be concluded that neither the dura nor the blood vessel significantly affect the recorded signal quality or performance.

Llinás, 2005 and Watanabe, 2009^32,33^ suggested the possibility of using a ‘nanoprobe’ that can endovascularly record the electrical activity of a single neuron, or small group of neurons. Thus far, work in endovascular neural interfaces suggests that high frequency brain signals^1,4,31^ such as signals in the high gamma range, fast ripples and high frequency oscillations are possible from electrodes placed on the surface of the brain, but the ability to record multiunit activity using the endovascular approach has not been shown. Two key factors that determine electrodes ability to record multiunit activity are electrode size and distance from neurons^36,54–56^. Modelling studies have predicted that electrodes <50 μm in diameter will be able to record brain signals in the range of local field potentials and multiunit or single unit spiking while larger electrodes can record fast ripples and cortical oscillations including cortical oscillations^36,43,47,57^. With small electrode, the “listening sphere” is small. Since recording from a neuron is a function of distance; the electric field falls as a square of the distance from the source (1/r^2^). This would imply that to record multiunit activity recording electrodes would need to be closer to 200 μm from target neurons^55,58–60^. The distance between the electrode within a blood vessel in the superior sagittal sinus was calculated between 260–680 μm. It could be speculated that high resolution recordings such as LFP’s may be theoretically possible if electrodes were small <50 μm in diameter and less than 200 μm from the region of interest. While theoretically possible to achieve multiunit recordings endovascularly, present generation devices will require significant design changes to achieve this. Furthermore, electrodes would need to be located within the cortical blood vessels, such as the central sulcal vein, where the distance between the electrode and the brain were was minimized to under 200 μm. Further work, should investigate the possibility of high resolution recording from the brain and the possibility of recording multiunit activity from within a blood vessel.

The SNR of the EV array is a strong indicator of decoder performance. The SNR of the signal was strongly correlated with offline decoder performance, with higher SNR resulting in greater decoding accuracy. As expected when SNR was close to 1, the decoding performance was close to chance. This is not surprising considering the many noise sources in neural recordings including thermal noise, movement artefacts, ambient electrical noise, etc. Results showed that the signal and the noise scale together, irrespective of the separate locations of implantation (SD, EV, and ED). Averaging trials has been used as one method of improving the SNR, however, in an ideal BMI it may not be viable to average multiple trials, as this will limit the speed at which assistive technologies can be commanded and controlled. Therefore, methods of improving SNR, such as active grounding, referencing, etc., should be considered more thoroughly.

The EV array is a potential minimally-invasive alternative to SD and ED as a brain-activated switch. The aim of a BMI is to create a bridge between the brain and the external world via prostheses. Recent work has shown the transforming nature of a brain-activated switch in one person who was completely locked-in by providing thought-controlled spellers and cursor control^9,61^. Brain-activated switch-type BMIs utilizing brain surface potentials, such as those obtained from SD and ED arrays, have emerged as a viable signal for long-term neural interfacing in BMI. Previous work with SD and ED arrays showed no difference in their abilities to decode neural signals^49^. Our results show that the signal quality, defined by the bandwidth of recording and the signal-to-noise ratio, is not significantly different between the ED, EV, and SD arrays. Our results also show that the decoding accuracy in detecting an evoked potential is also comparable between ED, EV, and SD arrays. The promising prior results from ED and SD arrays suggest that the EV array may be a viable location of BMI control that bypasses the need for a craniotomy. These results shed further light on the findings of Bower *et al*.^4^ who found SD and EV electrodes performed reliably and concluded similarities in the recordings. These results further motivate a clinical trial where movement intent may be decoded to control external devices such as a speller or a wheelchair.

Critical factors that affect decoding neural activity are the bandwidth of information, the distance from target neurons, density of electrodes, and noise in the recording. Regarding the quality of data recorded (Bandwidth and SNR), the EV array is comparable to ED and SD arrays. However, the coverage that can be achieved by present EV arrays is limited to a few centimetres and can only be near brain areas adjacent to sizeable blood vessels; the impact of these limitations remains to be evaluated. The spatial resolution in this study was limited by the inter**-**electrode distances and electrode sizes. Larger reductions in the electrode size could increase spatial resolutions and allow for the implantation of electrode arrays with greater electrode numbers.

Previous work in BMI showed that the medial wall along the superior sagittal sinus contains a wealth of information about movement and movement intent^62^. It would be ideal to resolve this information within the medial wall in the posterior parietal cortex, primary motor cortex, and supplementary motor area to control movement of external objects. The medial wall would be an ideal location for the location of the EV array in humans. With the spatial resolutions, presently achievable with the EV along with the signal quality, it would be feasible to achieve discrete control with a small number of electrodes within a blood vessel in the brain.

Recent technological advances have led to the emergence of endovascular arrays for chronic recording of brain signals. We have demonstrated that the quality of recordings from endovascular arrays is comparable to recordings from epidural and subdural arrays, with reduction in electrode size resulting in enhanced spatial resolution across all arrays. These findings indicate that the endovascular array provides hope for a minimally-invasive technique for recording neural activity from the brain without the need for craniotomy. Importantly, the finding that the performance of the endovascular array is comparable to subdural and epidural arrays provides support for a minimally-invasive brain sensor with potential for use in a closed-loop neuromodulation system, such as a brain-machine interface.

## Methods

### Animals

Six adult Corriedale ewes weighing 60–70 kg were used in this study. Experiments were conducted at The Florey Institute of Neuroscience and Mental Health and were approved by the Florey Institute Animal Ethics Committee. Studies were in accordance with the NHMRC Principles of Laboratory Animal Care, Prevention of Cruelty to Animals Act, Australia, 2004, and the NHMRC Australian Code of Practice for the Care & Use of Animals for Scientific Purpose (seventh edition, 2004).

Six animals were implanted with ED, EV, and SD arrays, however two of the SD and ED arrays and one of the EV arrays developed faults at the connector. Therefore, we evaluated the quality of recordings obtained from the ED (4 arrays), EV (5 arrays), and SD (4 arrays) arrays from six animals. As illustrated in Fig. 1, the ED and SD arrays were placed on different hemispheres and were adjacent to the EV array. The SD and ED arrays were manufactured by Cortec GMBH, Germany and the EV arrays were made in-house^1,25^. The SD and ED arrays comprised 24 electrodes, with eight electrodes each of 500 µm, 750 µm, and 1000 µm diameter. Inter-electrode distance between similar electrode sizes was 4 mm and adjacent dissimilar sizes was 1.5 mm. The EV array had two sizes of electrodes, 500 µm and 750 µm; inter-electrode distance varied between electrodes (~2–6 mm). All electrodes were made of platinum. In this study, we used three electrode sizes for the ED and SD arrays and two sizes for the EV array. The largest size on the ED and SD arrays of 1 mm diameter would not fit within the dimensions of the blood vessel to be implanted and so was not used on an EV array. The 1 mm diameter was used in the ED and SD arrays based on previous studies showing that this is the optimum size to improve noise susceptibility while maintaining acceptable spatial resolution for a brain-computer interface^36^. Device positions were assessed immediately after deployment, and prior to termination of the experiment.

### Surgery

Animals were administered antiplatelet medication (Aspirin, 100 mg) daily from two days prior to implantation to minimize thrombosis and this was continued until the termination of the experiment. To induce anaesthesia, animals were premedicated with sodium thiopentone followed by intubation and ventilation with Isoflurane in air/O_2_. A cut-down and direct cannulation of the jugular vein was followed by advancement of a coaxial catheter system into the superior sagittal sinus, adjacent to the motor cortex^1,25^. Implantation of the EV array was performed under visual guidance using digital subtraction angiography (Arcadis Avantic, Siemens, Munich, Germany) as reported previously^1,25^. Percutaneous leads of the arrays exited the skin at the back of the neck.

After a 3–4 days recovery period, the animals underwent a second surgery to implant the cortical surface arrays. Under anesthesia (Isoflurane), the SD and ED arrays were implanted via craniotomy (1.4 × 0.8 cm) over the motor and somatosensory areas (Fig. 1). The exposed dura was covered with silicone sheet and then with dental cement. Percutaneous leads of the arrays exited the skin at the back of the neck similar to the EV device. The animals were kept in individual pens for periods of 3–4 weeks, which we have shown is sufficient for all the electrodes to be incorporated into the tissue^1,25,63^.

Prior to termination of the animal, in an acute experiment, animals were pre-medicated with a bolus of Sodium Thiopental followed by maintenance with Propofol and ketamine. Following intubation, the animals were ventilated with air. The median nerve in the sheep was exposed and stimulating needle electrodes were implanted 5 mm apart, and cortical evoked potentials in response to median nerve stimulation were recorded. Both left and right legs were stimulated. Animals were euthanized with an overdose of pentobarbital.

### Cortical Recordings

Cortical electrophysiological signals (ECoG) were recorded using a g.tec USB amplifier (g.Tec, GMBH, Germany) at a sampling rate of 4800 Hz. ECoG recordings were analyzed using MATLAB (MathWorks Inc., Natwick, USA). Signals were band-pass filtered between 4–2400 Hz using a 4^th^ order butterworth filter and 50 Hz noise and associated harmonics were removed using a IIR comb filter. Broken electrodes were identified when the impedances were greater than 1 MΩ at 1 kHz and these were removed from the analysis. Artefacts, such as spikes due to the electronics or cable movement, were seen in awake recordings. Electrical artefacts were identified where the RMS amplitudes in 0.1 s segments with 0.05 s overlap between segments were greater than 20 times the average RMS amplitude of all electrodes of the array. Artefacts caused by chewing were identified using a Hilbert transform to obtain an envelope of the artefact and finding prominent peaks in the envelope. Segments with artefacts were visually verified and removed from further analysis. Recordings were common average re-referenced to the average of all electrodes of the same size on each array. For example, each 500 µm electrode within the EV array was common average referenced to all 500 µm electrodes on the EV array.

#### Signal Bandwidth

The bandwidth of the recorded signal is a meaningful indicator of electrode performance since ECoG follows a typical 1/f decrease in the signal amplitude ending in a flat response equal to the noise floor, where the signal is indistinguishable from the noise. To evaluate the bandwidth of recordings, the signals were recorded in awake animals 3 weeks after implantation. The animals were either standing or sitting in their pen with minimal interaction with the surroundings. Recordings were separated into 2 s windows and the power spectra were calculated for each electrode using the Thompson multitaper method with a centre frequency of 1 Hz (2 Hz resolution). The noise of each electrode was estimated using the spectral power between 800 Hz and 1200 Hz. This frequency band was chosen as it was the highest frequency band located below the Nyquist frequency and was where the asymptote of the 1/f spectral profile was clearly seen^1,42^. Regions of 10 Hz around each harmonic of 50 Hz were ignored in the analysis. Median spectral content in each 10 Hz bin (between 4 Hz to 1200 Hz) were compared to the noise estimate. A 10 Hz bin was considered dissimilar from noise if the median power was greater than the upper boundary of the noise (3^rd^ quartile + 1.5 IQR).

#### Signal-to-Noise Ratio (SNR)

The signal-to-noise ratio (SNR) offers a relative measure of quality of the recorded signal relative to the noise. We computed the SNR from electrically evoked cortical potentials by stimulating the median nerve in anaesthetized animals. Stimulation pulses were generated using a NI-myDAQ and LabView (National Instruments Corp., Austin USA) and passed through an AM-2200 stimulus isolator (AM systems, Sequim, USA).

Stimulation of the median nerve comprised constant current, monophasic, cathodal pulses between two electrodes placed 2 cm apart. Current amplitudes were varied randomly between 0 mA and 6 mA (0.5 mA steps). The maximum amplitudes used for stimulation varied between animals and were between 3 mA and 6 mA to reach maximum visible movement of the leg on stimulation. Stimuli were presented at 0.73 Hz and 10 repetitions were performed at each current level.

We measured the dependence of the SNR on the number of averaged trials for the three recording modalities. Averaging trials in electrophysiology is a strong tool, reducing the effect of noise with each additional trial. The slope (rate of change in the SNR with increasing number of averages) of the SNR, single trial SNR and 10 trial averaged SNR were expected to be different for varying array locations and electrode sizes. Both left and right limbs were stimulated, however only the stimulation with the lowest threshold of the two for each array was used in the SNR analysis. This enabled reducing any bias due where the stimulation was always contralateral to the one array while ipsilateral to another.

The evoked response to median nerve stimulation was analyzed by first segmenting the data into individual current levels and then averaging within each current level. The threshold current level of the evoked potential (*Th*) was detected by finding peaks in the data where the amplitude after the maximum stimulation ${\rm{\max }}\,{A}_{t0}^{t2}\,\,$was greater than twice the maximum amplitude of the background,

1 $$\begin{array}{c}Th=\,{\rm{\max }}\,{A}_{{t}_{0}}^{{t}_{200}} > 2\times \,{\rm{\max }}\,{A}_{{t}_{-200}}^{{t}_{0}},\end{array}$$

where, *t*_0_ was the stimulation time, *t*_-200_ was 200 ms prior to *t*0 and *t*2 was 200 ms after *t*_0_.

SNR was defined as the ratio of the root mean square (RMS) at threshold current level (*Th*) at time 100 ms (*t*_100_) after stimulation *t*_0_ (${{\rm{RMS}}}_{T{h}_{t0}}^{{t}_{100}}$) to the RMS at threshold current level (*Th*) at time 100 ms (*t*_−100_) before stimulation (*t*_0_) (${{\rm{RMS}}}_{T{h}_{{t}_{-100}}}^{t0}$),

2 $$SN{R}_{i}={{\rm{RMS}}}_{T{h}_{{t}_{0}}}^{{t}_{100}}/{{\rm{RMS}}}_{T{h}_{{t}_{-100}}}^{{t}_{0}}.$$

The SNR was calculated on the trial averaged signal in varying combinations of trials (*nCr*) without replication, where *n* is the total number of trials (10) and *r* is the number of trials (1, 2, 3, …, 10) used for each average. For example, C(1, 10) = 10 combinations in total i.e. SNR is calculated on single trials; C(2, 10) = 45 combination with 2 trials in each combination and SNR is calculated on each combination; C(5, 10) = 252 combinations with 5 trials in each combination and SNR is calculated on each combination and C(10,10) = 1 combination of 10 trials and SNR is calculated on the average of 10 trials.

### Spatial Resolution

Spatial resolution is a measure of the shared neural activity between adjacent electrodes. The spatial resolution achievable by neural recordings depends largely on the array location and size. To evaluate the effect of array location on spatial resolutions, we calculated the magnitude squared coherence^64^ between electrodes. Recordings were taken in awake animals while the animals were in their individual pens. Coherence is known to vary with distance^36,44^ that can be approximated by an exponential function ($Y=a{e}^{-bx}+c$)^39^. The function ‘Y’ approximates the rate of change ‘b’ of the coherence between electrode from a base constant of ‘a’ at distance ‘x’ and an offset of ‘c’. If the coefficient associated with b and/or d is negative, y represents exponential decay. The spatial resolution is approximated as the inter-electrode distance where magnitude squared coherence equals 0.3^36,39,44^.

In this study, we used magnitude squared coherence as a measure of spatial resolution during rest while the animal was awake and in its cage. In this period, the coherence would be dominated by the magnitude of the electric fields, which decrease as the inverse of the square of distance, and to a lesser extent functional connection in the brain. Since the correlation was not driven by an evoked stimulus, the correlation would be assumed to be mediated by the electrode’s ability to capture the change in the spatial localized brain rhythms. We measured the magnitude squared coherence in frequency bands relevant to motor induced oscillations in the brain (8–24, 25–80, 81–120, 121–180 Hz) as these are most commonly used in ECoG-based BCIs^65–67^. Magnitude squared coherence was calculated in frequency bands as previous work with ECoG showed that coherence is frequency dependent^39^ and provides a robust measure of signal quality for the SD, EV, and ED arrays.

### Evoked Potential Decoding

The decoding performance on the different arrays was assessed by classifying the presence or absence of an evoked potential using the recording features from cortical signals. All analysis was performed at the threshold current level identified earlier. Trials were bootstrap averaged in every combination of trials without replication (*nPr*), where *n* is the total number of trials (10) and *r* is the number of trials (1, 2, 3, …, 10) used for each average. We estimated the average power in the time in 4–24, 30–45, 55–95 and 105–145 Hz bands using Welch power estimate, on each trial combination. Evoked potential related features (i.e. power in the defined frequency band mentioned above) were extracted from all electrodes on each array and used to detect the presence of an evoked potential using a linear discriminant analysis and a leave half out cross validation where the classifier is trained on 50 percent of trials and then tested on the remaining 50 percent of trials. The process was repeated for every non-repeating combination and the average accuracy is reported.

### Statistics

Statistical tests were performed using MATLAB (MathWorks Inc., MA, USA) or Minitab (Minitab Inc.). Between groups comparison was made using ANOVA’s where assumptions of normality and variance were satisfied. Where assumptions of normality and variance were violated Kruskal-Wallis test was used when data distributions between groups were similar and Mood median test was if distribution shapes were different. Pearsons correlation analysis was used to analyse trends in the data and were defined weak (0–0.4), moderate (0.41–0.8) and strong (0.81–1.0).

### Data availability

The datasets generated during the current study are available from the corresponding author on reasonable request.

## References

[1] Oxley TJ (2016). Minimally invasive endovascular stent-electrode array for high-fidelity, chronic recordings of cortical neural activity. Nat. Biotechnol..

[2] Penn RD, Hilal SK, Michelsen WJ, Goldensohn ES, Driller J (1973). Intravascular intracranial EEG recording. Technical note. J. Neurosurg..

[3] Thomke F, Stoeter P, Stader D (1998). Endovascular electroencephalography during an intracarotid amobarbital test with simultaneous recordings from 16 electrodes. J. Neurol. Neurosurg. Psychiatry.

[4] Bower MR (2013). Intravenous recording of intracranial, broadband EEG. J. Neurosci. Methods.

[5] Sefcik RK (2016). The evolution of endovascular electroencephalography: historical perspective and future applications. Neurosurg. Focus.

[6] Yanagisawa T (2009). Neural decoding using gyral and intrasulcal electrocorticograms. Neuroimage.

[7] Schalk G (2007). Decoding two-dimensional movement trajectories using electrocorticographic signals in humans. J. Neural Eng..

[8] Miller, K. J. *et al*. Three cases of feature correlation in an electrocorticographic BCI. *Conf. Proc. IEEE Eng. Med. Biol. Soc*. **2008**, 5318–21 (2008). 10.1109/IEMBS.2008.4650415 19163918

[9] Vansteensel Mariska J., Pels Elmar G.M., Bleichner Martin G., Branco Mariana P., Denison Timothy, Freudenburg Zachary V., Gosselaar Peter, Leinders Sacha, Ottens Thomas H., Van Den Boom Max A., Van Rijen Peter C., Aarnoutse Erik J., Ramsey Nick F. (2016). Fully Implanted Brain–Computer Interface in a Locked-In Patient with ALS. New England Journal of Medicine.

[10] Williams JJ, Rouse AG, Thongpang S, Williams JC, Moran DW (2013). Differentiating closed-loop cortical intention from rest: building an asynchronous electrocorticographic BCI. J. Neural Eng..

[11] Martens S (2014). Epidural electrocorticography for monitoring of arousal in locked-in state. Front. Hum. Neurosci..

[12] Rouse AG, Williams JJ, Wheeler JJ, Moran DW (2013). Cortical adaptation to a chronic micro-electrocorticographic brain computer interface. J. Neurosci..

[13] Eliseyev A, Aksenova T (2014). Stable and artifact-resistant decoding of 3D hand trajectories from ECoG signals using the generalized additive model. J. Neural Eng..

[14] Shimoda K, Nagasaka Y, Chao ZC, Fujii N (2012). Decoding continuous three-dimensional hand trajectories from epidural electrocorticographic signals in Japanese macaques. J. Neural Eng..

[15] Yanagisawa T (2011). Real-time control of a prosthetic hand using human electrocorticography signals. J. Neurosurg..

[16] Milekovic T (2012). An online brain-machine interface using decoding of movement direction from the human electrocorticogram. J. Neural Eng..

[17] Tebo CC, Evins AI, Christos PJ, Kwon J, Schwartz TH (2014). Evolution of cranial epilepsy surgery complication rates: a 32-year systematic review and meta-analysis. J. Neurosurg..

[18] He BD, Ebrahimi M, Palafox L, Srinivasan L (2016). Signal quality of endovascular electroencephalography. J. Neural Eng..

[19] Boniface SJ, Antoun N (1997). Endovascular electroencephalography: the technique and its application during carotid amytal assessment. J. Neurol. Neurosurg. Psychiatry.

[20] Kara T (2014). Endovascular brain intervention and mapping in a dog experimental model using magnetically-guided micro-catheter technology. Biomed. Pap. Med. Fac. Univ. Palacky. Olomouc. Czech. Repub..

[21] Henz BD (2014). Advances in radiofrequency ablation of the cerebral cortex in primates using the venous system: Improvements for treating epilepsy with catheter ablation technology. Epilepsy Res..

[22] Henz BD (2008). Successful radiofrequency ablation of the cerebral cortex in pigs using the venous system: Possible implications for treating CNS disorders. Epilepsy Res..

[23] Opie NL (2016). Chronic impedance spectroscopy of an endovascular stent-electrode array. J. Neural Eng..

[24] Oxley, T. J. *et al*. An ovine model of cerebral catheter venography for implantation of an endovascular neural interface. *J. Neurosurg*. **128**, 1020-1027 (2018). 10.3171/2016.11.JNS161754 28452616

[25] Opie Nicholas Lachlan, van der Nagel Nicole R., John Sam E., Vessey Kirstan, Rind Gil S., Ronayne Stephen M., Fletcher Erica L., May Clive N., O'Brien Terence J., Oxley Thomas J. (2017). Micro-CT and Histological Evaluation of an Neural Interface Implanted Within a Blood Vessel. IEEE Transactions on Biomedical Engineering.

[26] Wong, Y. T. *et al*. Suitability of nitinol electrodes in neural prostheses such as endovascular neural interfaces. In *2016 38th Annual International Conference of the IEEE Engineering in Medicine and Biology Society (EMBC) 4*463–4466 (IEEE, 2016). 10.1109/EMBC.2016.7591718 28269269

[27] Nakase H (1995). An intra-arterial electrode for intracranial electro-encephalogram recordings. Acta Neurochir. (Wien)..

[28] Stoeter P, Dieterle L, Meyer a, Prey N (1995). Intracranial electroencephalographic and evoked-potential recording from intravascular guide wires. AJNR. Am. J. Neuroradiol..

[29] Mikuni N (1997). ‘Cavernous sinus EEG’: a new method for the preoperative evaluation of temporal lobe epilepsy. Epilepsia.

[30] Kunieda T (2000). Use of Cavernous Sinus EEG in the Detection of Seizure Onset and Spread in Mesial Temporal Lobe Epilepsy. Epilepsia.

[31] Ishida S (1998). Intracranial EEG Recording from Intravascular Electrodes in Patients with Temporal Lobe Epilepsy. Epilepsia.

[32] Llinás RR, Walton KD, Nakao M, Hunter I, Anquetil PA (2005). Neuro-vascular central nervous recording/stimulating system: Using nanotechnology probes. J. Nanoparticle Res..

[33] Watanabe H, Takahashi H, Nakao M, Walton K, Llinás RR (2009). Intravascular Neural Interface with Nanowire Electrode. Electron. Commun. Japan.

[34] Buzsáki G, Anastassiou CA, Koch C (2012). The origin of extracellular fields and currents–EEG, ECoG, LFP and spikes. Nat. Rev. Neurosci..

[35] Weiner GM, Ozpinar A, Ducruet A (2016). Endovascular Access for Cortical Mapping. Neurosurgery.

[36] Wodlinger, B., Degenhart, A. D., Collinger, J. L., Tyler-Kabara, E. C. & Wang, W. The impact of electrode characteristics on electrocorticography (ECoG). *Conf. Proc. IEEE Eng. Med. Biol. Soc*. **2011**, 3083–6 (2011). 10.1109/IEMBS.2011.6090842 22254991

[37] Rohde MM (2002). Quality estimation of subdurally recorded, event-related potentials based on signal-to-noise ratio. IEEE Trans. Biomed. Eng..

[38] Lahr J (2015). Invasive brain–machine interfaces: a survey of paralyzed patients’ attitudes, knowledge and methods of information retrieval. J. Neural Eng..

[39] Muller L, Hamilton LS, Edwards E, Bouchard KE, Chang EF (2016). Spatial resolution dependence on spectral frequency in human speech cortex electrocorticography. J. Neural Eng..

[40] Gunduz A, Sanchez JC, Carney PR, Principe JC (2009). Mapping broadband electrocorticographic recordings to two-dimensional hand trajectories in humans Motor control features. Neural Netw..

[41] Warren DJ (2016). Recording and Decoding for Neural Prostheses. Proc. IEEE.

[42] Bundy DT (2014). Characterization of the effects of the human dura on macro- and micro-electrocorticographic recordings. J. Neural Eng..

[43] Wilson JA, Felton EA, Garell PC, Schalk G, Williams JC (2006). ECoG factors underlying multimodal control of a brain-computer interface. IEEE Trans. Neural Syst. Rehabil. Eng..

[44] Wang, W. *et al*. Human motor cortical activity recorded with Micro-ECoG electrodes, during individual finger movements. In *Annual International Conference of the IEEE Engineering in Medicine and Biology Society* **2009**, 586–9 (2009). 10.1109/IEMBS.2009.5333704 PMC3142578 19964229

[45] Wong, Y. T. *et al*. Suitability of nitinol electrodes in neural prostheses such as endovascular neural interfaces. In *Proceedings of the Annual International Conference of the IEEE Engineering in Medicine and Biology Society, EMBS* **2016–Octob** (2016). 10.1109/EMBC.2016.7591718 28269269

[46] Rousseau H (1987). Self-expanding endovascular prosthesis: an experimental study. Radiology.

[47] Slutzky MW, Jordan LR, Miller LE (2008). Optimal spatial resolution of epidural and subdural electrode arrays for brain-machine interface applications. Conf. Proc. IEEE Eng. Med. Biol. Soc..

[48] Baek D-H (2014). A thin film polyimide mesh microelectrode for chronic epidural electrocorticography recording with enhanced contactability. J. Neural Eng..

[49] Flint RD, Rosenow JM, Tate MC, Slutzky MW (2017). Continuous decoding of human grasp kinematics using epidural and subdural signals. J. Neural Eng..

[50] Degenhart AD (2016). Histological evaluation of a chronicallyimplanted electrocorticographic electrode grid in a non-human primate. J. Neural Eng..

[51] Vistnes LM, Ksander GA, Kosek J (1978). Study of encapsulation of Silicone Rubber Implants in Animals. Plast. Reconstr. Surg..

[52] Gabriel S, Lau RW, Gabriel C (1996). The dielectric properties of biological tissues: III. Parametric models for the dielectric spectrum of tissues. Phys. Med. Biol..

[53] Gabriel C, Peyman A, Grant EH (2009). Electrical conductivity of tissue at frequencies below 1 MHz. Phys. Med. Biol..

[54] Rubehn B, Bosman C, Oostenveld R, Fries P, Stieglitz T (2009). A MEMS-based flexible multichannel ECoG-electrode array. J. Neural Eng..

[55] Khodagholy D (2015). NeuroGrid: recording action potentials from the surface of the brain. Nat Neurosci.

[56] Kim, J., Wilson, J. A. & Williams, J. C. A Cortical Recording Platform Utilizing µECoG Electrode Arrays. In *2007 29th Annual International Conference of the IEEE Engineering in Medicine and Biology Society* **2721**, 5353–5357 (IEEE, 2007). 10.1109/IEMBS.2007.4353551 18003217

[57] Wang X (2017). Mapping the fine structure of cortical activity with different micro-ECoG electrode array geometries. J. Neural Eng..

[58] Einevoll GT, Kayser C, Logothetis NK, Panzeri S (2013). Modelling and analysis of local field potentials for studying the function of cortical circuits. Nat. Rev. Neurosci..

[59] Lindén H (2011). Modeling the spatial reach of the LFP. Neuron.

[60] Kajikawa Y, Schroeder CE (2011). How local is the local field potential?. Neuron.

[61] Moritz CT, Perlmutter SI, Fetz EE (2008). Direct control of paralysed muscles by cortical neurons. Nature.

[62] Yoo PE (2018). 7T-fMRI: Faster temporal resolution yields optimal BOLD sensitivity for functional network imaging specifically at high spatial resolution. Neuroimage.

[63] Sillay KA (2013). Long-term measurement of impedance in chronically implanted depth and subdural electrodes during responsive neurostimulation in humans. Brain Stimul..

[64] Kay, S. M. *Modern Spectral Estimation*. *Book* (Prentice-Hall, 1988).

[65] Collinger JL (2014). Motor-related brain activity during action observation: a neural substrate for electrocorticographic brain-computer interfaces after spinal cord injury. Front. Integr. Neurosci..

[66] Morris S (2014). Patient Specific Cortical Electrodes for Sulcal and Gyral Implantation. IEEE Trans. Biomed. Eng..

[67] Ball T, Schulze-Bonhage A, Aertsen A, Mehring C (2009). Differential representation of arm movement direction in relation to cortical anatomy and function. J. Neural Eng..

