# Author Correction: Signal quality of simultaneously recorded endovascular, subdural and epidural signals are comparable

**DOI:** 10.1038/s41598-018-36257-8

**Published:** 2018-11-27

**Authors:** Sam E. John, Nicholas L. Opie, Yan T. Wong, Gil S. Rind, Stephen M. Ronayne, Giulia Gerboni, Sebastien H. Bauquier, Terence J. O’Brien, Clive N. May, David B. Grayden, Thomas J. Oxley

**Affiliations:** 1 Department of Biomedical Engineering, The University of Melbourne, Parkville, Australia; 2 Vascular Bionics Laboratory, Department of Medicine, Royal Melbourne Hospital, (RMH), The University of Melbourne, Parkville, Australia; 3 Florey Institute of Neuroscience and Mental Health, Parkville, Australia; 4 Centre for Neural Engineering, The University of Melbourne, Carlton, Australia; 5 Department of Veterinary Science, The University of Melbourne, Werribee, Australia; 6 Department of Physiology and Department of Electrical and Computer Systems Engineering, Monash University, Clayton, Australia; 7 SmartStent Pty Ltd, Parkville, Australia

Correction to: *Scientific Reports* 10.1038/s41598-018-26457-7, published online 30 May 2018

This Article contains an error in the Y axis of Figure 4a, where ‘0.2’ and ‘0.4’ should read ‘0.5’ and ‘1.0’ respectively. The correct Figure 4 appears below as Figure 1.

**Figure 1:**
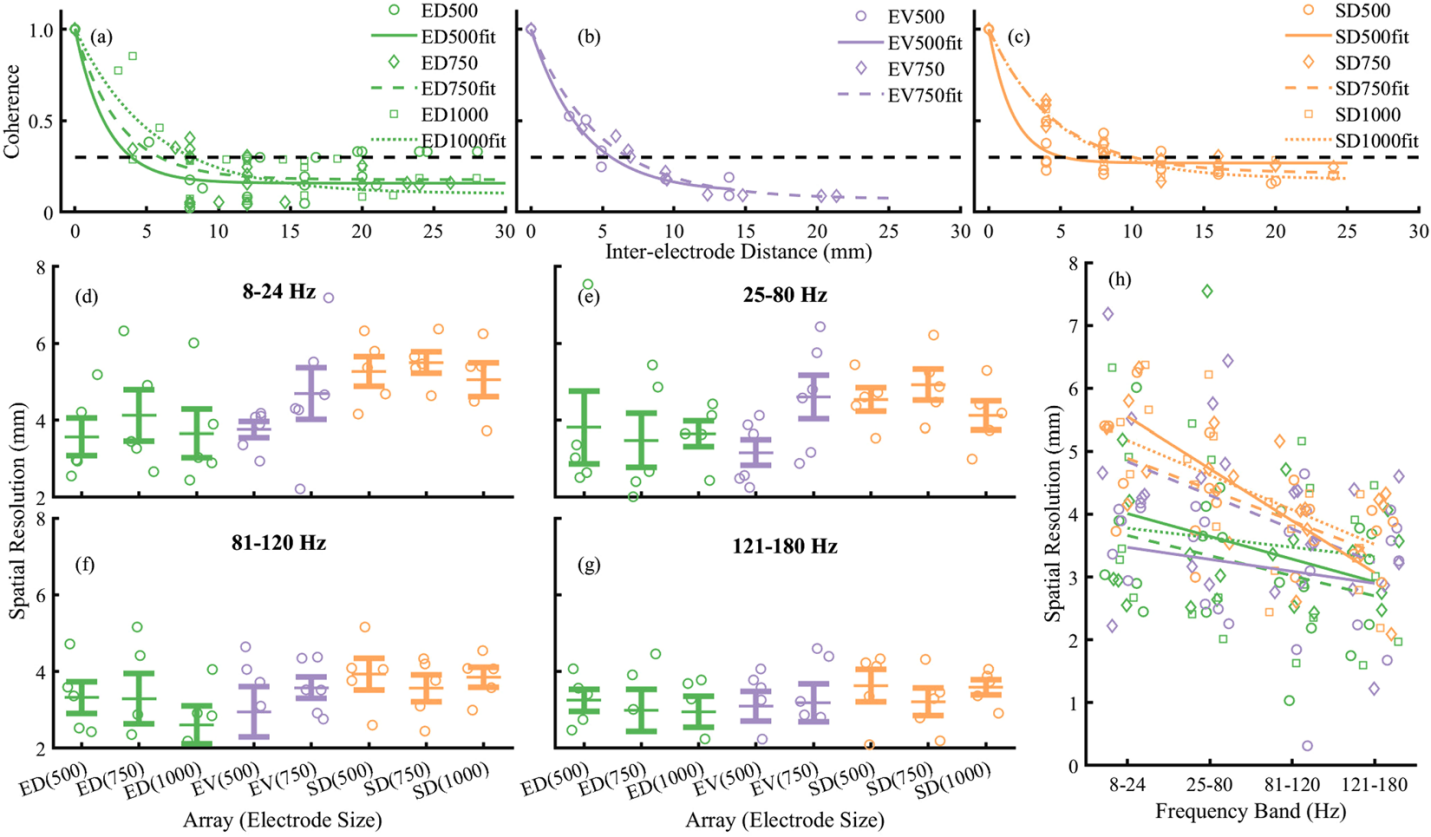
Spatial resolution. Representative data showing the estimation of spatial resolution using magnitude squared coherence versus the inter-electrode distance for (**a**) ED, (**b**) EV, and (**c**) SD electrodes. Fits were estimated as an exponential function of the magnitude squared coherence and were weighted to the inverse of the inter-electrode distance. The dashed horizontal line at 0.3 shows the level at which the signals between signals were considered independent. (**d**) Spatial resolutions at 8–24 Hz. (**e**) Spatial resolutions at 25–80 Hz. (**f**) Spatial resolutions at 81–120 Hz. (**g**) Spatial resolutions at 121–180 Hz. Symbols show individual values, centre lines show mean, error bars show standard error of the mean. Kruskal-Wallis test showed a significant effect of electrode location in the low frequency (**d**) (p = 0.003). However, there was effect of electrode size at any frequency band (p > 0.05) or electrode location at frequencies greater than 24 Hz (**e**–**g**). (**h**) Frequency dependence of spatial resolution, symbols indicate electrode size and lines are global fits at each electrode size and array. Pearson’s correlation analysis showed strong negative correlation between spatial resolution and frequency for all electrode sizes with SD arrays ρ > 0.6 (p < 0.05); moderate negative correlation 750 μm diameter EV electrodes ρ = 0.45 (p < 0.05); and weak correlations not significantly different to zero for ED electrodes and 500 μm diameter EV electrodes (p > 0.1).

